# Sequencing of breast cancer stem cell populations indicates a dynamic conversion between differentiation states *in vivo*

**DOI:** 10.1186/bcr3687

**Published:** 2014-07-06

**Authors:** Daniel Klevebring, Gustaf Rosin, Ran Ma, Johan Lindberg, Kamila Czene, Juha Kere, Irma Fredriksson, Jonas Bergh, Johan Hartman

**Affiliations:** 1 Department of Medical Epidemiology and Biostatistics, Karolinska Institutet, Stockholm, Sweden; 2 Science For Life Laboratory, Stockholm, Sweden; 3 Department of Biosciences and Nutrition, Center for Biotechnology, Karolinska Institutet, Stockholm, Sweden; 4 Department of Oncology-Pathology, Karolinska Institutet, Stockholm, Sweden; 5 Research Programs Unit, University of Helsinki, and Folkhälsan Institutet of Genetics, Helsinki, Finland; 6 Department of Molecular Medicine and Surgery, Karolinska Institutet, Stockholm, Sweden; 7 Department of Breast and Endocrine Surgery, Karolinska University Hospital, Stockholm, Sweden; 8 Radiumhemmet – Karolinska Oncology, Karolinska University Hospital, Stockholm, Sweden; 9 Department of Clinical Pathology, Karolinska University Hospital, Stockholm, Sweden

## Abstract

**Introduction:**

The cancer stem cell model implies a hierarchical organization within breast tumors maintained by cancer stem-like cells (CSCs). Accordingly, CSCs are a subpopulation of cancer cells with capacity for self-renewal, differentiation and tumor initiation. These cells can be isolated through the phenotypic markers CD44+/CD24-, expression of ALDH1 and an ability to form nonadherent, multicellular spheres *in vitro*. However, controversies to describe the stem cell model exist; it is unclear whether the tumorigenicity of CSCs *in vivo* is solely a proxy for a certain genotype. Moreover, *in vivo* evidence is lacking to fully define the reversibility of CSC differentiation.

**Methods:**

In order to answer these questions, we undertook exome sequencing of CSCs from 12 breast cancer patients, along with paired primary tumor samples. As suggested by stem classical cell biology, we assumed that the number of mutations in the CSC subpopulation should be lower and distinct compared to the differentiated tumor cells with higher proliferation.

**Results:**

Our analysis revealed that the majority of somatic mutations are shared between CSCs and bulk primary tumor, with similar frequencies in the two.

**Conclusions:**

The data presented here exclude the possibility that CSCs are only a phenotypic consequence of certain somatic mutations, that is a distinct and non-reversible population of cells. In addition, our results imply that CSCs must be a population of cells that can dynamically switch from differentiated tumor cells, and vice versa. This finding increases our understanding of CSC function in tumor heterogeneity and the importance of identifying drugs to counter de-differentiation rather than targeting CSCs.

## Introduction

Breast cancer is a heterogeneous disease reflected at both a morphological and genetic level [1,2]. Earlier described as a simple chaotic expansion of clonal subpopulations of cancer cells, breast cancer now appears as a hierarchical organization of cells. Cancer cells within the primary tumor exist in a variety of differentiation states and with different mutations, some specific to certain subclones. In conformity with the structural organization of the normal mammary gland, cancer cells with stem cell characteristics (CSC) have been suggested to reside in the apex of this evolutionary tree [3]. CSCs were first discovered in leukemia where their existence revolutionized the view on malignant hematological diseases [4]. Accordingly, only a small subset of cancer cells with a distinct cell-surface glycoprotein profile was able to initiate leukemia in mouse transplantation assays, now considered the gold standard for CSC characterization. Since then, CSCs have been identified in several solid tumor types such as melanoma [5], colorectal [6] and breast cancer [7]. Most probably as a consequence of their proliferative quiescence, CSCs have been shown to be more resistant to chemotherapy [8]. Therefore, the presence of dormant breast CSCs has been proposed as an explanation for the late relapse of breast cancer. Although frequently debated, according to the classical CSC hypothesis, the CSC is converted from a normal stem cell through gradual accumulation of genetic alterations [9]. As a consequence of their self-renewal capacity, CSCs are able to maintain tumor growth over long periods of time. By asymmetrical cell division, the slow-proliferating CSCs seed progenitor cells with high proliferative capacity. These cells will differentiate and constitute the bulk of the tumor, although each cell has a limited life span [10,11]. In the normal mammary gland as well as in the hematopoietic systems, the majority of stem cells are dormant and cells within the more differentiated states should have no possibilities to turn back into the parental stem cell state.

We postulate that a similar scenario exists in breast cancer. Accordingly, mutations within the slowly dividing mammary CSC should be of driver character and essential for both early tumor initiating events and tumor propagation. The majority of passenger mutations must instead be gained in the differentiated cancer cell state, where each round of cell division may initiate new mutations. We here refer to this model as the classical stem cell (SC) model.

In recent years a contrasting scenario of the hierarchical organization has been proposed; plasticity within tumor cells enables differentiated cancer cells to reverse into a CSC state through epigenetic events. This would enable any epithelial cell to initiate a tumor through acquisition of a SC phenotype, here referred to as the plasticity model. The existence of such a plasticity scenario has been shown *in vitro* by cell line experiments and theoretical models. However, the hypothesis has not been validated in a more clinical setting [12,13].

Mammary CSCs are characterized by their tumor initiating capacity in xenograft models. This capacity is highly enriched within the subpopulation of cancer cells forming multicellular spheres during non-adherent conditions, called mammospheres [14,15]. Moreover, sphere formation correlates to high expression of embryonal SC genes, in turn correlating to poor prognosis in breast cancer. Another way to isolate mammary CSCs is through the selection of cells with certain membrane markers. Cancer cells of the CD44+/CD24- phenotype and with aldehyde dehydrogenase 1 (ALDH1^High^) activity have been repeatedly shown to possess SC characteristics [7,10]. As shown by mouse transplantation assays, the tumor initiating capacity specific for the CSC population indicates an irreversible conversion from CSC to its progeny cells. If not, the differentiated cancer cells would switch back into a CSC state and also be able to initiate tumor growth.

As a consequence of the proposed difference in the mutational spectrum in CSC and differentiated cells of the two models, we sought to investigate this by mutational profiling of the different cancer cell populations. We identified a striking genetic similarity of the two cell populations that, indeed, strongly indicates a dynamic fluctuation of cancer cells between the CSC and differentiated states *in vivo*.

## Methods

From ten patients, cells from bulk tumor were enriched for mammospheres according to detailed procedures described in Additional file 1. After 14 days of culture, the cells were divided into two replicates, each subjected to whole-genome amplification and handled separately in downstream library preparation. The replication was carried out in order to distinguish amplification artifacts from the whole genome amplification from true somatic mutations. From two additional patients, CD44+/24- and ALDH1^High^ CSC populations were isolated by fluorescent activated cell sorting (FACS). From the same patients, DNA was extracted from whole blood and CD44- and ALDH1^Low^ cells, representing differentiated tumor cells. All samples were subjected to exome sequencing. In brief, DNA was fragmented using ultrasonication and barcoded adapters were ligated. The four barcoded libraries from each patient were pooled and subjected to sequence capture of 63,564,965 bases corresponding to 242,233 exons and miRNAs in the human genome. Somatic variants were identified and a list of shared, mammosphere-specific and tumor-specific mutation was compiled for each patient while discarding amplification artifacts.

All patients participating in the study signed an informed consent according to the Declaration of Helsinki. The study was approved by the Regional Ethical Review Board in Stockholm, Sweden.

## Results

**Figure 1:**
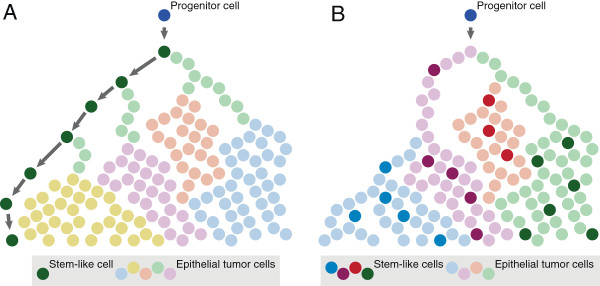
**Schematic overview of the two main hypotheses of breast cancer stem cell hierarchy. (A)** Cancer stem cells irreversibly convert into to progenitor cells. The cancer stem cells have the possibility of asymmetrical division, producing a new cancer stem cell and a tumor cell, whereas differentiated tumor cells do not. Changing colors represent the introduction of somatic events in the tumor. Arrows between stem-like cancer cells denote a slow turnover rate. The much higher turnover rate of differentiated tumor cells means that mutations that arise in those cells by far would outnumber the ones that originate in stem cells. **(B)** Dynamic state between cancer stem cells and differentiated tumor cells, where interconversions between differentiation-states is possible. The rapid turnover of differentiated tumor cells yields a spread of mutations across both the bulk tumor and cancer stem cells.

As described above and in Figure 1, two alternative and opposing explanations have been proposed to describe the tumorigenic process governed by CSCs in the mammary gland. In the classical SC model (A), we expect an accumulation of unique mutations with low allelic frequencies in the non-SCs as a consequence of high proliferation and irreversible conversions from the SC to the differentiated state. Within the plasticity model (B), there is a dynamic and reversible differentiation in the tumor and the CSC state may be adopted by any cancer epithelial cell through epigenetic de-differentiation [12,13]. Accordingly, mutations and their allelic frequencies should not differ in the two compartments.

In order to investigate the two contradicting hypothesis of CSCs, we isolated and propagated mammospheres from the primary breast tumors by directly transferring single sphere forming cells from tumor biopsies. This method has been shown to propagate CSC whereas differentiated cells cannot survive the non-adherent conditions. We performed exome sequencing of the bulk primary tumor (consisting of >70% epithelial tumor cells in all the ten patients), paired normal DNA from leukocytes and mammospheres to examine the somatic mutational patterns in CSCs and bulk primary tumor for each patient (outline of procedures in Additional file 1: Figure S1 and S2). To further validate the CSC phenotype of mammospheres, they were assessed by immunofluoresence imaging and about 70% to 80% of cells were identified as CD44+/CD24- and more than 85% were ALDH1^High^ (Additional file 1: Figure S3). Furthermore, OCT4, SOX2 and NANOG were expressed in mammospheres, but not in primary tumors consistent with a SC cell phenotype (Additional file 1: Figure S4).

With exome sequencing, we detected on average 99 somatic mutations in the exome of the bulk primary tumor cells and 98 mutations in the CSCs of the 10 patients, which is in line with previous data on somatic mutations in breast cancer [16,17]. Out of the top ten mutated genes in the large breast cancer investigation of The Cancer Genome Atlas (TCGA) [16], we found mutations in seven of these (PIK3CA, GATA3, MAP3K1, MLL3, CDH1, PTEN, TBX3). Notably, none of the ten patients in this study harbored somatic mutations in TP53, even though it is the most commonly mutated gene in breast cancer. Most somatic TP53 mutations occur in patients with the triple negative (TN) or human epithelial growth factor receptor 2 (HER2) subtypes (TN: tumors without expression of the estrogen and progesterone receptors and lacking overexpression of HER2 (ErbB2); HER2: tumor with overexpression of HER2 [16]. Of the ten patients included in our study, only two had tumors of the TN subtype and one of the HER2 subtype. No single mutation was found in more than one patient (all somatic mutations are available in Additional file 2).

**Figure 2:**
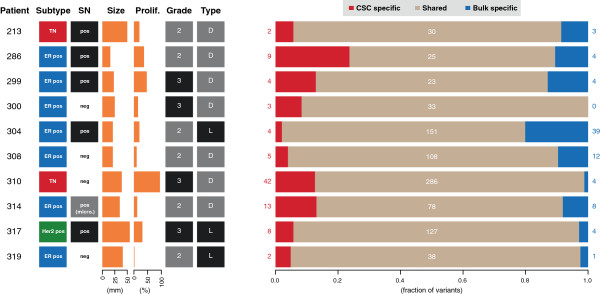
**Histopathological characteristics and fraction of unique and shared mutations between cancer stem cells and bulk tumors.** No association of the fraction of unique or shared mutations with any histopathological characteristics were found. Subtype classification according to immunohistochemical staining: TN (ER-, PR-, HER2-), ER pos (ER+, PR+/-, HER2-), HER2 pos (ER-, PR-, HER2+). SN: Sentinel node metastasis positive or negative. Size in mm. Proliferation according to percentage of Ki67/MIB1 positive cells. Grade according to the pathology assessment. D, Ductal; L, Lobular type. Red, beige and blue numbers indicate the fraction of mutations unique to the cancer stem cells, shared and unique to the bulk tumor, respectively. Numbers next to and in the bars of the rightmost part of the plot indicate the number of mutations in each category. ER, estrogen receptor; HER2, human epithelial growth factor receptor 2; PR, progesterone receptor; TN, triple negative.

**Figure 3:**
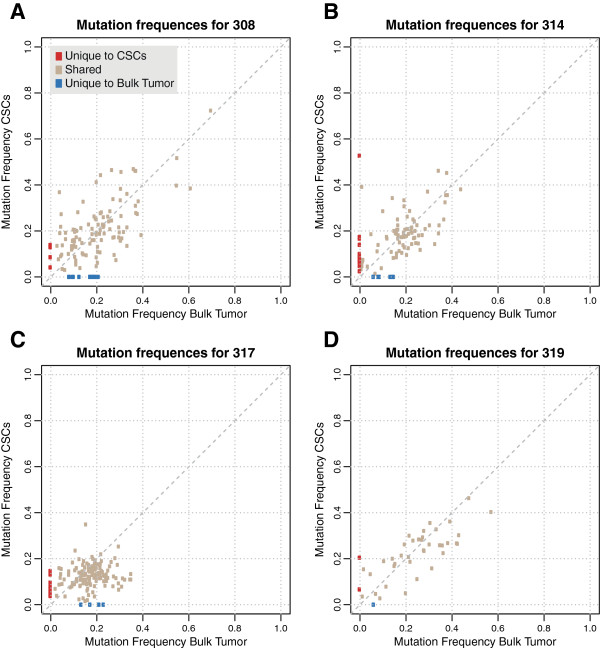
**Allele frequencies for unique and shared mutations in four patients.** Mutation allele frequencies for patients **A)** 308, **B)** 314, **C)** 317 and **D)** 319. A wide spread across frequencies is detected, with similar frequencies in stem cells and bulk tumor. Shared mutations are shown in beige, mutations unique to the cancer stem cells are shown in red whereas those unique to the bulk tumor are shown in blue. Plots for all patients in the study can be found in Additional file 1: Figure S5.

We investigated the extent of shared mutations between CSCs and bulk primary tumor cells. On average, 83% of the mutations were shared (range 65% to 93%, Figure 2). No significant association between the number of shared mutations and the total number of mutations in each tumor was found (*P* = 0.71; *Student’s* t-test). To study the association between the number of shared mutations and tumor characteristics we collected relevant data from the histopathology reports, including data on tumor type (lobular or ductal), size, grade (according to Elston-Ellis), lymph node status, estrogen and progesterone receptor (ER and PR) status, presence of HER2 amplification and proliferation (Ki-67/MIB1). For tumor type, size, grade and proliferation, the spread of the variables was enough to mandate statistical analysis versus the percentage of shared mutations. Both uni- and multivariate variant analyses were performed, but no significant differences were detected. We investigated the mutated allele frequency of the shared mutations in the CSCs and the primary tumor cells. The median mutation allele frequency across all patients was 17% (90% of the frequencies were between 3.2% and 50%). The frequency of each mutation was similar between the CSCs and bulk tumor (Figure 3; Additional file 1: Figure S5).

We also compared the allele frequencies of mutations unique to the bulk primary tumor cells and CSCs to those that were shared. To achieve enough power, we performed statistical testing only where at least five unique mutations in either CSC or bulk primary tumor cells were available. In the ten patients included, we performed eight tests. In all tests except one, the allele frequencies of the unique mutations in CSC or bulk primary tumor cells were significantly lower (*P* <0.05) than those of the shared mutations.

In order to validate the somatic mutations found in exome sequencing, we also performed ultra-deep amplicon sequencing across fourteen mutation sites in three of the patients. Of these fourteen mutations, three were shared according to the original data as well as the validation data. Five mutations were unique to the bulk primary tumor cells in the exome data. Of these, four were shared in the validation data, whereas one was a false positive in the original exome data. Six mutations were unique to the CSCs in the exome data. Here, one was validated as CSC-unique, albeit at a low allele frequency (5%). The remaining five mutations were either shared (one mutation), false positive calls or technical artifacts in the exome data (three and one mutations, respectively). Thus, we conclude that the mutations found to be unique in either the CSCs or bulk primary tumor cells were, when re-sequenced with ultra deep coverage, in fact validated as shared between the two compartments, or false positives in the original exome sequencing.

By exome sequencing of paired CSCs and bulk primary tumor cells we show that the somatic mutations in the differentiated epithelial cells and CSCs are highly similar, and that the allele frequencies are spread over the spectrum. After validation with ultra deep re-sequencing of fourteen mutations discovered in the original exome sequencing we found only one mutation to be unique in the CSCs and that in a low frequency (4.8%). The tumor tissue used for isolation of mammospheres and DNA preparation was taken from the same 10 mm core biopsies (Additional file 1: Figure S2) but a mutation in a local clone present only in the tissue used for mammosphere isolation might be an explanation for this finding.

In the next step, we sought to perform a similar investigation based on two alternative methods to isolate CSC. By FACS of fresh tumor biopsies, we isolated the CD44+/CD24- subpopulation from one tumor and ALDH1^High^ cells from a second tumor. The two different CSC subpopulations were exome sequenced along with corresponding non-CSC (CD44- and ALDH1^Low^) populations. Leukocytes from the two patients were used as control for germline mutations. For the patient where ALDH1 was used to isolate CSC, the somatic mutations were identical in ALDH1^High^ and ALDH1^Low^ populations with similar mutation frequencies in the two (Additional file 1: Figure S6). When using two markers for selection (CD44+/CD24-), the yield of cells was considerably low, around 1% of the total input (approximately 3,000 cells). In order to investigate the status of mutations identified in the CD44- population, we counted the total number of reads that support the variant allele across all mutation positions within CD44-, CD44+/CD24- and the leukocyte samples. For the CD44-, 21.6% (967 of 4,476) of all reads support the variant allele. For the CD44+/CD24- and leukocytes, these numbers were 17.5% (81 of 462) and 0.4% (24 of 5,354), respectively. This clearly indicates that the mutations present in the CD44- populations are also present in the selected CD44+/CD24- subpopulation (Additional file 1: Figure S7).

## Discussion

The large-scale DNA sequencing efforts in recent years have provided us with an enormous amount of data pointing at a substantial intra- and intertumoral genetic diversity in breast cancer. Although mutations in certain classes of genes such as PI3K are frequent, no mutations common for all breast tumors have been identified. It is not clear whether the intratumoral genetic diversity also correlates to differentiation states within tumor cells. CSCs have been detected by immunohistochemistry markers in the majority of breast cancers and they seem to be heterogeneously situated in the tumor [18]. Consequently, in the classical SC model, phenotypic differences, such as described with the CSC model, could reflect genetic differences. If so, the tumorigenicity of the proposed CSC population will be a result of a certain clonal expansion. The classical SC model would also indicate a similar scenario of non-shared mutations between the CSC subpopulation and the differentiated progeny cell. Evidence provided by experiments on cancer cell lines *in vitro* are now indicating plasticity within the CSC model and a reversible transition of cancer cells between differentiation states [12,13]. Recently, Balic *et al.* performed a DNA profiling investigation by comparative genomic hybridization (CGH) array on FACS sorted MCF7 and SUM159 cell lines based on CD44/24 and ALDH1to evaluate cancer cell heterogeneity [19]. However, cell lines are clonally homogenous and are not recapitulating the complex tumor microenvironment *in vivo*. Shipitsin and colleagues performed copy number analysis using DNA microarrays of CD44+ versus CD24+ cell populations from a single breast cancer patient and showed that both populations shared copy number alterations, indicating a clonal origin [20]. The same group later showed a similar genetic diversity between CD44+ and CD24+ cell populations for a subset of genetic markers commonly altered in breast cancer [21]. Taken together, these studies point to reversible transitions being a common mechanism in mammary CSC differentiation, where genetic alterations are propagated to reach similar frequencies in the different subpopulations. In that case, genome-wide investigations on purified CSC populations from patient-derived tumors would be an essential step forward to provide further evidence of this concept.

We undertook this project in two steps. First, we examined the genetic similarity of the CSC population selected by mammosphere formation versus bulk tumor DNA from the corresponding breast tumor. Since only cells with tumor initiating capacity are able to overcome anoikis and to initiate and maintain mammopshere growth, we decided to use this method for CSC selection. Single cells were plated into non-adherent cell-culture dishes in selective cell medium. We then confirmed the SC phenotype of the propagated mammospheres by immunofluorescence imaging and FACS based on CD44/24 and ALDH1. However, it is impossible to completely rule out the risk of contamination in the mammospheres by de-differentiation of cancer cells into CSC during artificial *in vitro* conditions. We, therefore, performed a second step of genetic comparison, now by directly selecting CSCs by the SC markers CD44+/24- and ALDH1^High^. The mutational spectrum of the CSC population was then compared with the corresponding differentiated tumor cell populations.

The data presented by us show that the CSC and differentiated tumor cells are genetically very similar. Hence, intratumoral genetic diversity cannot explain the tumorigenicity of the CSC population. Our results instead show a common genetic background for CSCs and the differentiated tumor cells and point to a dynamic and reversible transition between stem-like and differentiated cells in the tumor [12,13]. If the CSCs would represent a distinct lineage of cells, or be a cell-of-origin of the differentiated cells (as illustrated in Figure 1A), one would, due to the low rate of cell-division [22] and strict hierarchy, have expected the number of mutations in the CSC subpopulation to be lower compared to the differentiated tumor cells. Since the differentiated cancer cells have a high proliferation rate and express higher levels of proliferation related markers compared to the CSCs [22] they would generate a far larger number of mutations, and with a minor or absent degree of shared mutations. In contrast, we see a large degree of shared mutations. A dynamic cell-state model (Figure 1B), where interconversion between cellular phenotypes occurs, would better explain both this large degree of shared mutations and the spread in frequencies seen in both CSCs and bulk primary tumor. Even though investigation of the allele frequencies alone does not enable distinct classification into different subclones, it provides strong evidence that early mutations (present at high frequencies) are propagated across cell states in early tumorigenesis and therefore are present in similar frequencies across different cell populations. As mutations occur in the rapidly dividing epithelial cells, they are propagated to daughter cells that are occasionally converted to stem-like cells also harboring the mutation. We, therefore, argue that the CSCs are not on the apex of the hierarchy but rather exist in parallel to the differentiated cells, with differentiation and de-differentiation being common features within a solid tumor. These results are in contrast to hematopoietic cancers, where a clear SC origin has been established [23,24].

## Conclusions

The fact that the vast majority of mutations are shared between CSCs and the bulk primary tumor together with the observed distribution of allele frequencies indicates that a dynamic transition between cellular states takes place continuously throughout the tumor development. The dynamic transitions between the CSC and differentiated state suggested here have important implications for therapy. In order to completely eradicate the tumor and obliterate any risk of a late relapse, therapy should not be focused on only one component, but instead involve therapies targeting both subpopulations and especially preventing de-differentiation into a CSC state.

## Abbreviations

CSC: cancer stem cell; ER: estrogen receptor; FACS: fluorescent activated cell sorting; HER2: human epithelial growth factor receptor 2; miRNA: microRNA; PR: progesterone receptor; SC: stem cell; TCGA: The Cancer Genome Atlas; TN: triple negative.

## Competing interests

The authors declare that they have no competing interests.

## Authors’ contributions

JH and DK conceived of the study. DK, KC, JB, JK and JH designed the experiments. IF performed biospecimen collection and obtained informed consents. GR and RM performed laboratory procedures, FACS-sorting and mammosphere cultures. DK and JL analyzed the data. All authors critically interpreted the data. DK, GR and JH drafted the paper. All authors read and approved the final manuscript.

## Supplementary Material

Additional file 1 Additional Figures S1–S7, Additional Tables S1–S2 and Full materials and methods. Click here for file

Additional file 2 The full set of somatic variants detected in this study, as output from MuTect. Click here for file
