# Supplementary material for: Sequencing of breast cancer stem cell populations indicates a dynamic conversion between differentiation states in vivo
Source: Breast Cancer Res. 2014 Jul 6;16(4):R72. doi: 10.1186/bcr3687 (PMC4227057; doi:10.1186/bcr3687)
Supplement: Additional file 1 — Additional Figures S1–S7, Additional Tables S1–S2 and Full materials and methods. [file bcr3687-S1.docx]

Additional Materials

**Additional Figure 1**


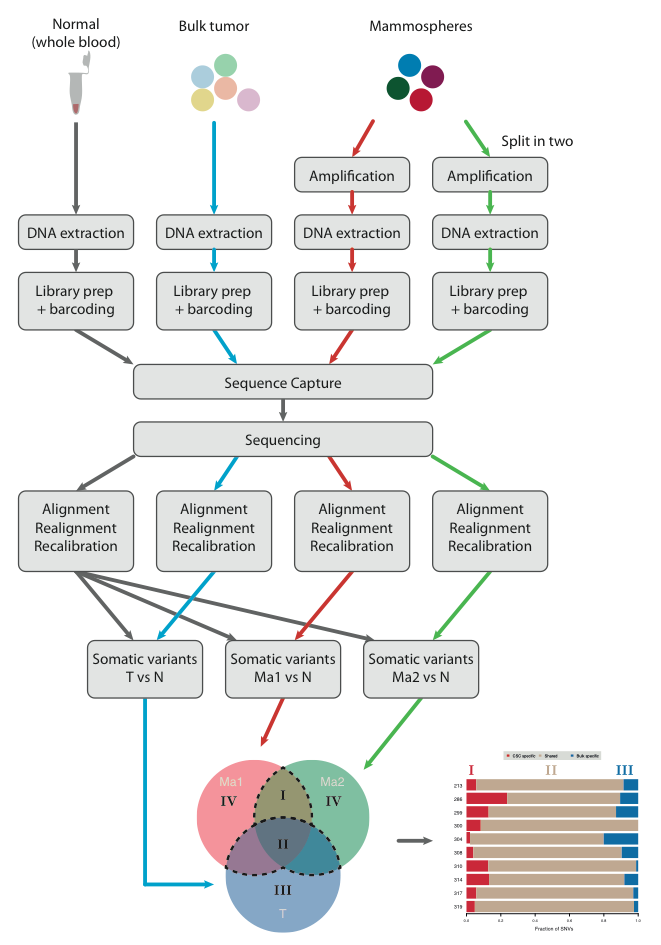


**Additional Fig. 1**. Outline of the workflow of the study. DNA from mammospheres was amplified in replicates in order to minimize amplification artifacts. For each patient, a set of 4 barcoded samples (normal, N, bulk tumor, T, two mammosphere replicates, Ma1, Ma2) was sequenced together. Variants for each of the three relevant contrasts (T vs. N, Ma1 vs. N, Ma2 vs. N) were called and compared to produce lists of shared and unique variants.

**Additional Figure 2**


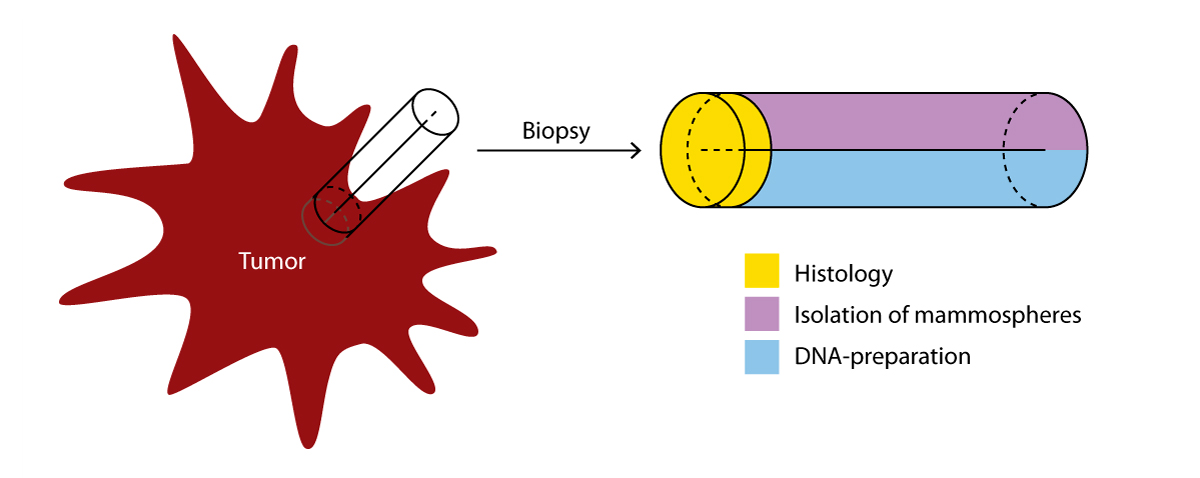


**Additional Fig. 2**. From each tumor, a 10 mm core biopsy was punched. The biopsy was then divided into three different parts; one for histology and calculation of tumor cell density, one for whole-tumor DNA preparation and one for immediate mammosphere isolation.

**Additional Figure 3**

**Additional Fig. 3.** (**A**) Mammospheres derived from primary breast cancers were separated and transferred as single cells onto cell-culture dishes and further propagated. Representative bright-field images of first (G1) and second generation mammospheres (G2; Day 1-7). (**B**) Immunofluorescence imaging of mammospheres with antibodies for CD44 (green), CD24 (red) and nuclei counterstained with 4′,6-diamidino-2-phenylindole (DAPI, blue). (**C**) Single sphere-initiating cells were labeled with PKH26 (yellow) and then left to proliferate for 1 week (n=5). Counterstaining of nuclei with DAPI (blue). All mammospheres were positive for PKH26 but the intensity varied across individual cells within spheres. (**D**) ALDH1 staining (green) together with DAPI counterstaining of nuclei. In newly formed mammospheres, all cells were ALDH1^High^ whereas larger mammospheres were partially ALDH1^High^. (**E**) Mammospheres derived from primary breast cancers were separated into single-cells and labeled with CD44-FITC and CD24-PE; 44% of cells were CD44^+/^CD24^-^.

**Additional Figure 4**


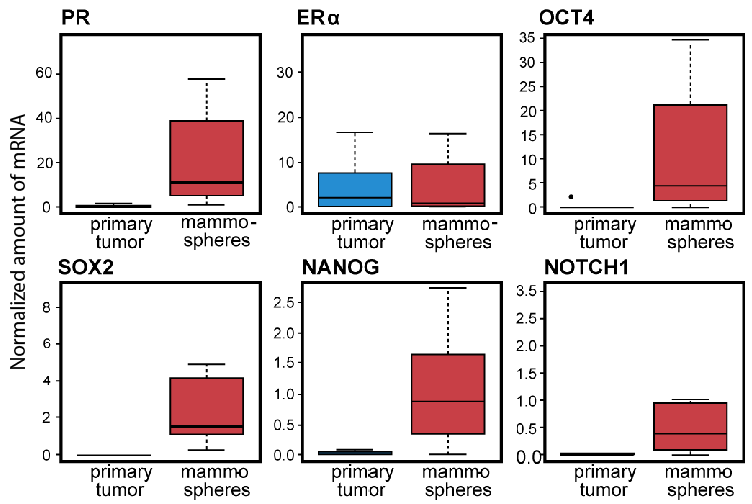


**Additional Fig. 4.** Characterization of the mammospheres and bulk primary tumor by qRT-PCR from 20 patients. The of classical pluripotency genes OCT4, SOX2, NANOG and NOTCH1 are highly expressed in the mammospheres, but practically unexpressed in the differentiated tumor cells.

**Additional Figure 5**

**
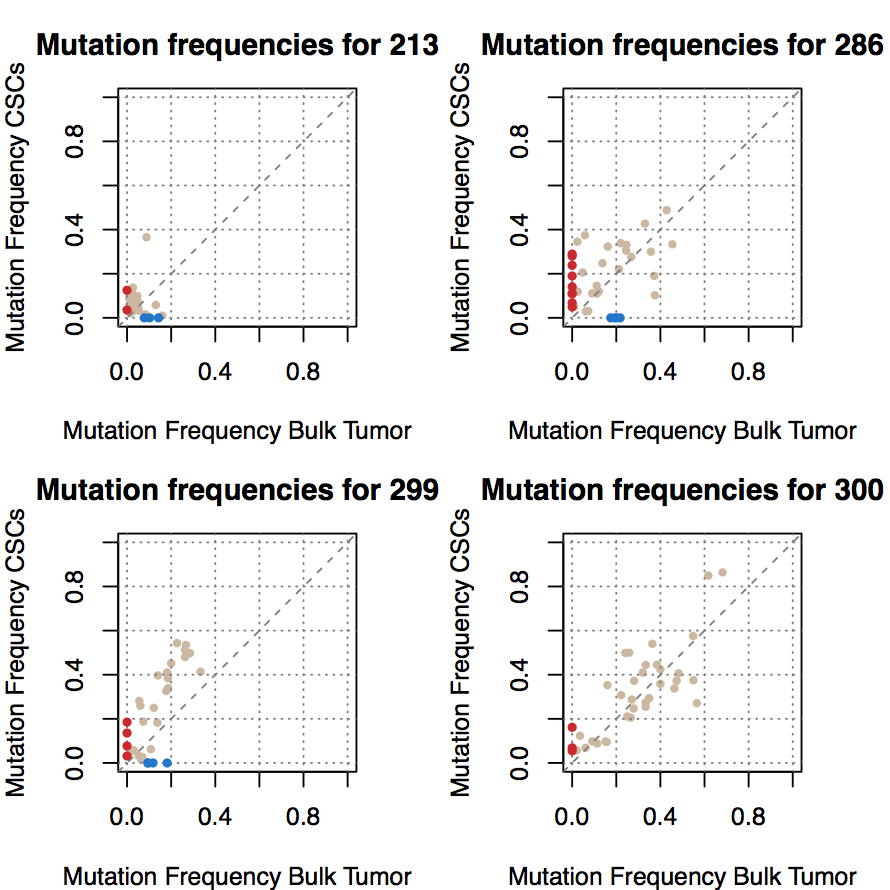
**

**
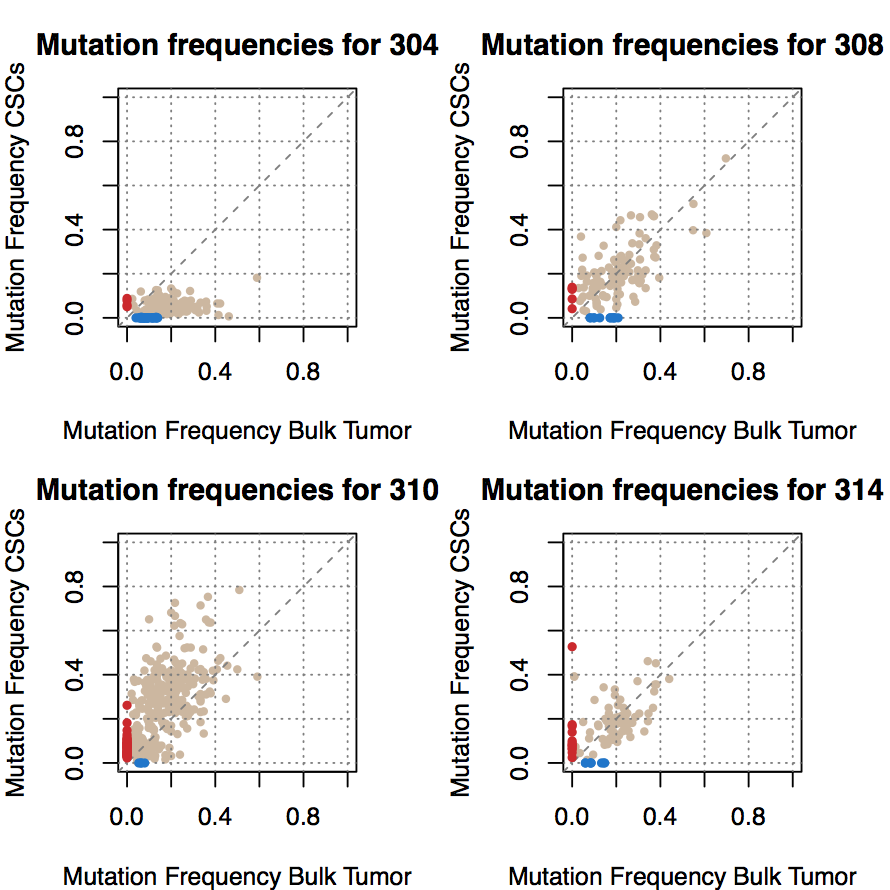
**

**
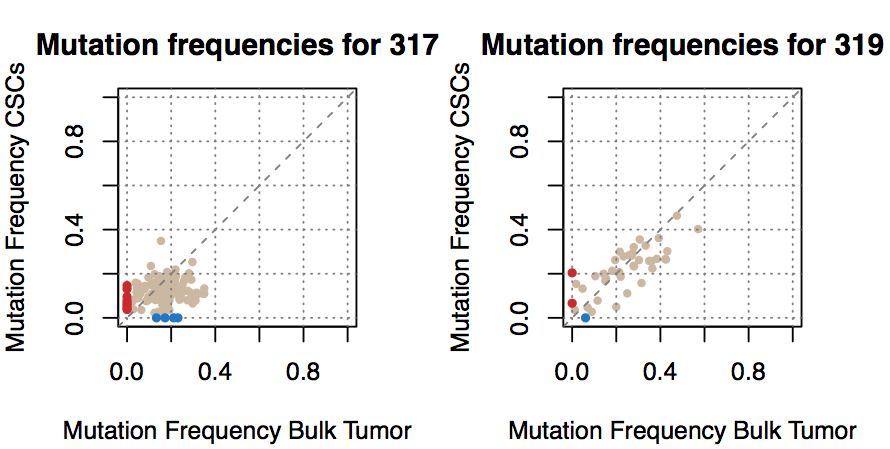
**

**Additional Fig. 5.** Allele frequencies for unique and shared mutations in all patients. A wide spread across frequencies is detected, with similar frequencies in stem cells and bulk tumor. Shared mutations are show in beige, mutations unique to the CSCs are shown in red whereas those unique to the bulk tumor are shown in blue.

**Additional Figure 6**

**
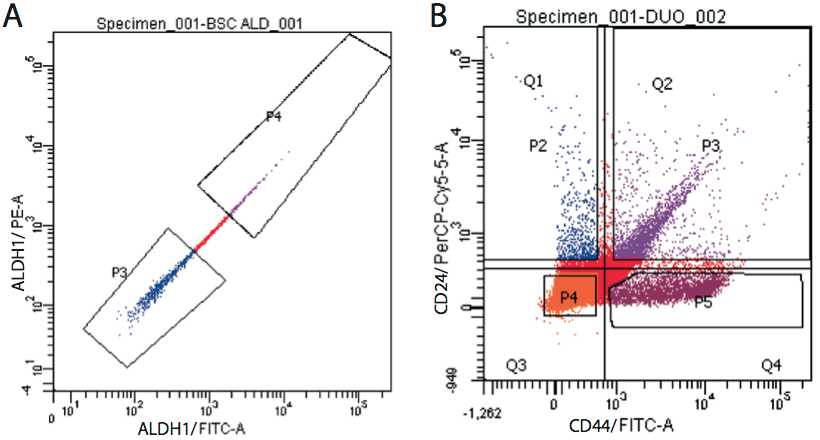
**

**Additional Figure 6. (A)** ALDH1 positive cells in gate P4 were selected as stem-like cells for sequencing and P3 as bulk tumor cells. In total, P4 corresponded to approximately NN (15% of total) and P3 of NN (50% of total) cells. (B) CD44+/CD24- cells in gate P5 were selected as stem-like cells for sequencing and combined P2 and P4 were used as bulk tumor. The gate P5 contained ≈3000 cells (1% of total), combined P4 and P2 273,000 cells (94% of total).

**Additional Figure 7**


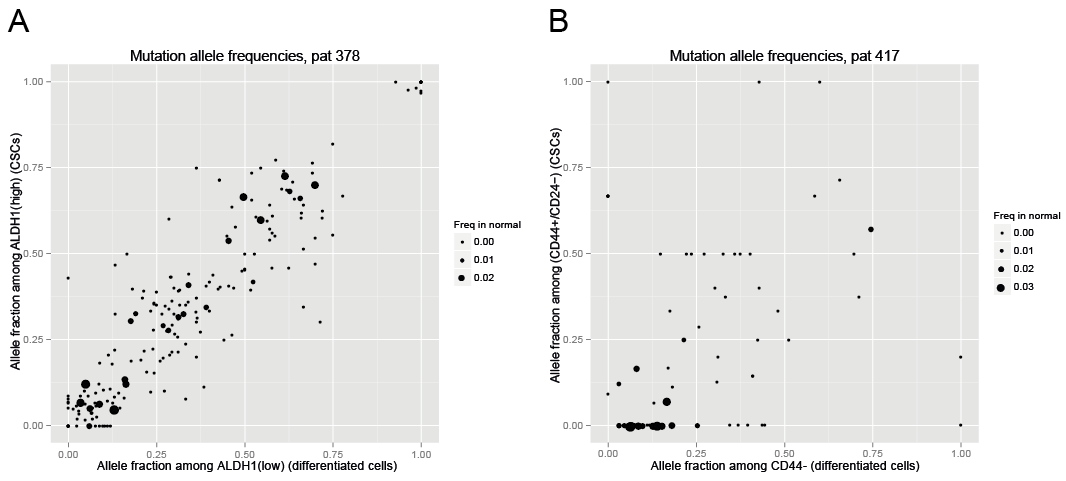


**Additional Figure 7.** Mutational spectrum for patients 378 and 417, where cells were sorted by ALDH1 status (A) and CD24/CD44 (B) status, respectively. For the CD44+/CD24- cells of patient 417, the yield was low causing low DNA extraction yield and thus low coverage (approximately 5x). Only variants at a high frequency in the CD44- cells could therefore be detected in the CSC population.

**Additional Table 1**

Somatic mutation calls from MuTect for each sample.

**Additional Table 2**

Clinico-pathological characterstics for the patients included in the study

| **Selection Method** | **ID** | **HER2 IHC** | **HER2 FISH** | **Ki67 Prolif** | **ER** | **PR** | **Size (mm)** | **Elston** | **Type** | **SN** |
| --- | --- | --- | --- | --- | --- | --- | --- | --- | --- | --- |
| Mammospheres | **310** | 0 | np | 60% | 0% | 0% | 39 | 3 | Ductal | NEG |
| Mammospheres | **304** | 0 | np | 20% | 95% | 95% | 21 | 2 | Lobular | POS |
| Mammospheres | **300** | 1 | np | 15% | 95% | 80% | 25 | 3 | Ductal | NEG |
| Mammospheres | **286** | 1-2+ | NEG | 37% | 80% | 90% | 16 | 2 | Ductal | POS |
| Mammospheres | **299** | 0 | np | 47% | >95% | >95% | 23 | 3 | Ductal | POS |
| Mammospheres | **317** | 3+ | POS | 31% | 0% | 0% | 55 | 3 | Lobular | POS |
| Mammospheres | **314** | 1+ | np | 12% | 95% | 95% | 35 | 2 | Ductal | POS (micro) |
| Mammospheres | **319** | 0 | np | 2% | 90% | 90% | 41 | 2 | Lobular | NEG |
| Mammospheres | **213** | 0 | np | 20% | 5% | 5% | 50 | 2 | Ductal | POS |
| Mammospheres | **308** | 2+ | NEG | 10% | 95% | <1% | 21 | 2 | Ductal | NEG |
| FACS ALDH1 | **644** | 0 | np | 80% | 0% | 0% | 26 | 3 | Ductal | NEG |
| FACS CD44/24 | **671** | 0 | np | 20% | 100% | 100% | 25 | 2 | Ductal | NEG |
| ER, estrogen receptor; PR, progesterone receptor; SN, sentinel node; NEG, negative; POS, positive; np, not performed  Elston, Elston grade | | | | | | | | | | |

**Materials and Methods**

**Enrichment of stem-like cells from bulk tumor and subsequent formation**

Fresh primary breast cancer tumor tissue and blood were collected at the Karolinska University Hospital, Stockholm, Sweden during 2011 and 2012. All tissues were obtained by written informed consent and in compliance with standardized surgical procedures approved by the regional ethical board. After collection the tissue specimen was immediately processed for purification of mammospheres. In short, cells were seeded at 2x103 cells per well in 6-well Ultralow Adherence plates (Corning Inc., Corning, NY) in MammoCultTM Proliferation Supplements in proportion with MammoCultTM Basal Medium (1:10) (STEMCELL Technologies Inc., Canada), 4 μg/mL heparin solution, 0.48 μg/mL hydrocortisone, and 1% penicillin (streptomycin), modified from manufacturer’s protocol (STEMCELL Technologies). After 7 days, mammospheres were collected by centrifugation, washed with PBS, dissociated to single cells with tripLETM Express (GIBCO®) using pasteur pipet, and seeded to obtain the next generation of mammospheres, again after 7 days. For each generation of mammospheres, colonies were counted and their size evaluated after 3 days of incubation using the GelCountTM and its software (Oxford Optronix Ltd., Oxford, United Kingdom).

**Immunofluorescence**

The mammospheres were cytospun onto glass slides, fixed using 4% paraformaldehyde (PFA) during 20 min, blocked in PBS with 0.05% tween and bovine serum albumin for 45 min (all from Sigma-Aldrich). The primary antibodies used to characterize the immunophenotype of the mammospheres were: rabbit polyclonal anti-human CD44 1:100 (Sigma HPA005785), mouse monoclonal, anti-humanCD24-biotin (clone: 32D1) 1:50 (StemCell TECHNOLOGIES, 10231), rabbit polyclonal anti-human ALDH1 1:100 (Abcam, ab23375). For PKH26-staining, cells were incubated with 10-7M PKH26 (Sigma-Aldrich) for 5 min and then grown in suspension for 7 days to enable the formation of new spheres. Stained samples were analyzed and digital images were taken under a computerized fluorescence microscope equipped with CCD camera. Pictures were processed with Photoshop CS5. Mammospheres were characterized and images presented in Figure S3.

**Separation of the ALDH-Positive Population by FACS Sorting**

Primary cells obtained from freshly dissociated breast cancer patient biopsy were isolated with their ALDH enzymatic activities by using the ALDEFLUOR kit (StemCell Technologies, Grenoble, France). In general, around 50,000 cells were suspended in 500ul ALDEFLUOR assay buffer with 5ul ALDH substrate (BAAA) and incubated critically at 37°C for 40min. As the negative background control, a spare of an aliquot cells from the same biopsy were co-treated with ALDH substrate and equal volume of an ALDH inhibitor diethylaminobenzaldehyde (DEAB) during incubation. The positive sorting gate was identified using the negative-control cells stained with propidium iodide.

**FACS sorting procedures for the CD44+/CD24- population**

Single cells trypsinized from primary mammospheres were labeled with PE-conjugated Mouse Anti-Human CD24 (clone: SN3) and FITC-conjugated Mouse Anti-Human CD44 (clone: MEM-85), and propidium iodide (BD Pharmigen) analyzed by fluorescence-activated cell sorting (FACS) according to the manufacturer’s protocols.

**RT-qPCR**

RNA from mammospheres were collected and isolated, cDNA-synthesized and amplified with MessageBOOSTER^TM^ and Cell Lysates Kit (Epicentre biotechnologies, Madison, WI) in accordance with the manufacturer’s instructions. Bulk tumor RNA was first purified with RNeasy MinElute Cleanup Kit (QIAGEN) and then processed as above, all according to manufacturers instructions.

**DNA extraction**

Germline DNA was extracted from 400 µl whole-blood using FlexiGene DNA kit (Qiagen) following manufacturer’s instructions. Fresh frozen tumor tissue (approx. 3x3x3 mm3) was homogenized using Minilys (Precellys) 3 x 10 seconds at 5000 RPM in Qiagen buffer ATL. Twenty microliters of Qiagen proteinase K was added and incubated at 56 °C for 2 h with vortexing every 20 min. Clean up was carried out using QIAamp DNA micro (Qiagen) with an elution volume of 30 µl. Amplified mammosphere DNA was cleaned up using spin columns following the manufacturer’s instructions (QiaQiuck, Qiagen). DNA concentration was determined using Qubit according to manufacturers instructions (Invitrogen/Life Technologies).

**Library preparation**

Five hundred ng of DNA was adjusted to 120 µl using 0.1x EB and fragmented to a target average length of 300 bp using sonication on a Covaris S2 instrument with the following parameters: Duty cycle: 10%; Intensity: 4; Cycles per burst: 200; Duration: 120 s. The volume was adjusted to 50 µl using SpeedVac and used as input to TruSeq DNA Sample Preparation following the manufacturers instructions (TruSeq DNA Sample Preparation Guide, Part # 15026486 Rev. C, Illumina). Barcoding was performed using sets of four barcodes that were compatible in sequencing based on their sequence composition. After the ligation step, remaining free adapters were removed using size-selective precipitation on carboxylic acid­-coated superparamagnetic beads as previously described ^1^. Amplification by PCR was carried out according the library preparation protocol (Illumina) after which an additional clean up on beads was performed.

For the selected cells, 50 ng genomic DNA was fragmented as above and used as input for ThruPLEX-FD Prep kit (Rubicon Genomics) according to the manufacturers protocol.

**Sequence Capture**

Two hundred and fifty ng of library from tumor, normal, and each two amplified mammosphere replicates (each with a different barcode as described above) were pooled and subjected to multiplexed sequence capture using SeqCap EZ Exome version 3.0 following manufacturers instructions (NimbleGen SeqCap EZ Library SR User's Guide, Version 3.0) with the modification that the blocker “TS-INV-HE Index Oligo” was changed to equal amounts of four blockers each targeting one of the barcodes used for the samples. For patients used for FACS selection, 166 ng of each library (ALDH1+, bulk tumor, leukocytes for patient 378 and CD44+CD24-, bulk tumor and leukocytes for patient 417) for a total of 1 µg was used for capture as above. The total amount of blocker was kept at 1000 pmol. Multiplexed sequencing was carried out using Illumina Hiseq 2000 as instructed by the manufacturer (Illumina) yielding on average 90 million reads per sample after demultiplexing.

**Alignment**

Alignment of raw data to the human genome hg19 was performed using bwa version 0.6.2 ^2^ with the following parameters: -e 20 -q 10 -t 8. Amplification duplicate were removed using Picard MarkDuplicates version 1.63 with standard parameters ^3^. On average, 20% of the reads were discarded as PCR duplicates yielding an average of 71 million reads per sample. Local realignment and base quality recalibration was performed using the Genome Analysis Toolkit version 1.6.4 ^4^. We performed quality control using Picard’s CollectMultipleMetrics and CalculateHsMetrics and noted a slightly larger fold-80 base penalty in the amplified samples over the non-amplified (4.08 vs. 2.45, respectively). The average target coverage across all samples was 53x.

**Calling of somatic variants**

For each patient, somatic mutations between tumor and normal, as well as somatic mutations between each of the two mammosphere samples and normal, were called using MuTect version 1.0.27783 ^5^. Only positions with coverage over 14 in the tumor or mammosphere sample, and 8 in the normal, were investigated. Variants that were in regions of low alignability or in simple repeats were removed.

Due to the fact that somatic mutation can occur in only a few cells, there is a risk that a mutation is not called properly at low to moderate coverage. Therefore, for each patient, we assembled a list of all somatic mutations from the bulk tumor and two mammosphere replicates. This list was then used to calculate the number of reads supporting the alternative base, and the reference base in each of the three components (bulk tumor and mammosphere replicates).

In order to estimate a technical background, we calculated the “allele frequency” supporting either of the two bases other than the reference and called somatic mutation for each patient. This yielded a distribution “technical error”. In order to significantly say that a mutation was present in a sample we required the mutated allele frequency to be larger than the 95-percentile of the technical background.

**Detection of shared variants**

We identified mutations using the following scheme (categories refer to the different parts of the Venn diagram in Additional Figure S1):

Mutations shared by both mammosphere replicates but not present in the tumor represents mammosphere-specific mutations (category I, median 7, range 3-69).

Mutations shared between the bulk tumor and mammospheres were identified as those being present in the tumor sample and at least one of the two mammosphere replicates (category II, median 79, range 10-435).

Mutations present in the bulk tumor but in neither of the two mammosphere replicates represent tumor-specific mutations (category III, median 7, range 2-50)

Mutations present in only one of the two mammosphere replicates and not the bulk tumor were excluded as amplification artifacts (category IV, median 16, range 3 to 51).

Alternative allele frequencies from the tumors were calculated as the number of reads supporting the mutation divided by the total read depth in that position. For the mammospheres, this was calculated by adding the number of reads supporting the mutation in both mammosphere replicates and dividing by the sum of the read depth in both replicates.

**Validations**

A set of 14 somatic mutations across three patients (5 tumor-specific, 6 mammosphere-specific and 3 shared) was selected for validation using ultra-deep amplicon sequencing as described earlier ^6^. Compared to previous literature, we chose to increase the amount of genomic DNA of each reaction from 300-600 copies ^6^ to ≈ 6000 haploids copied in order to archive maximum depth for each sample. Amplification by PCR was carried out on DNA from the tumor, normal and each of the two whole-genome amplified mammosphere replicates separately.

PCR primers for each site were designed using primer3 through the prrimer3 R-package ^7^. The design was performed so that the amplicon length was between 80 and 150 bp, and with other settings left at default (including target Tm at 60°C). Amplification by PCR was carried out in a total volume of 20 µl per reaction with the following contents: 10 pmol forward primer, 10 pmol reverse primer, 20 ng of genomic DNA (corresponding to ≈ 6000 haploid copies) and 10 µl Phusion PCR mix (Finnzymes). The cycling conditions were 98 °C for 30 s, followed by 35 cycles of 98°C for 10 s, 65°C for 15 s and 72°C for 15 s. A final extension was performed at 72°C for 10 min.

PCR products were run on a 1% agarose gel in order to verify the fragment size, after which four pools were assembled; a tumor pool, a normal pool and one pool from each of the two mammosphere replicates. One microgram of DNA from each pool was used as input for a library preparation using the TruSeq kit as instructed by the manufacturer (Illumina) with different indexes. Sequencing was carried out by spiking a 2x150 bp paired-end MiSeq run (performed as instructed by the manufacturer, Illumina) with 2.5% from each of the four libraries. In order to take advantage of the paired-end sequencing strategy, read pairs were subjected to the SeqPrep software ^8^ which merges overlapping sequences into virtual single-end reads and thereby increases the base qualities in the overlap. The alignment strategy outlined above was used, with the exception that single-end (merged paired-end reads) reads aligned. When creating multiple-pileup files, the coverage was limited to 1000x in each position.

References:

1. Lundin, S., Stranneheim, H., Pettersson, E., Klevebring, D. & Lundeberg, J. Increased throughput by parallelization of library preparation for massive sequencing. *PLoS ONE* **5,** e10029 (2010).

2. Li, H. & Durbin, R. Fast and accurate short read alignment with Burrows-Wheeler transform. *Bioinformatics* **25,** 1754–1760 (2009).

3. *Picard*. at <http://picard.sourceforge.net>

4. McKenna, A. *et al.* The Genome Analysis Toolkit: A MapReduce framework for analyzing next-generation DNA sequencing data. *Genome Research* **20,** 1297–1303 (2010).

5. Cibulskis, K. *et al.* Sensitive detection of somatic point mutations in impure and heterogeneous cancer samples. *Nat Biotechnol* (2013). doi:10.1038/nbt.2514

6. Shah, S. P. *et al.* The clonal and mutational evolution spectrum of primary triple-negative breast cancers. *Nature* (2012). doi:10.1038/nature10933

7. *Prrimer3*. (2012). at <https://bitbucket.org/dakl/prrimer3>

8. *SeqPrep*. (2012). at <https://github.com/jstjohn/SeqPrep>
